# A study of the correlation between STEM career knowledge, mathematics self-efficacy, career interests, and career activities on the likelihood of pursuing a STEM career among middle school students

**DOI:** 10.1186/s40594-018-0118-3

**Published:** 2018-05-16

**Authors:** Karen A. Blotnicky, Tamara Franz-Odendaal, Frederick French, Phillip Joy

**Affiliations:** 1 Department of Business Administration and Tourism and Hospitality Management, Mount Saint Vincent University, Halifax, NS B3M 2J6 Canada; 2 Department of Biology, Mount Saint Vincent University, Halifax, NS B3M 2J6 Canada; 3 Department of Education, Mount Saint Vincent University, Halifax, NS B3M 2J6 Canada; 4 School of Health and Human Performance, Dalhousie University, Halifax, NS B3H 4R2 Canada

**Keywords:** STEM career, Mathematics self-efficacy, Technical skills, Career knowledge, Career awareness, Career interests, Career activities, Education, Subject requirements

## Abstract

**Background:**

A sample of 1448 students in grades 7 and 9 was drawn from public schools in Atlantic Canada to explore students’ knowledge of science and mathematics requirements for science, technology, engineering, and mathematics (STEM) careers. Also explored were their mathematics self-efficacy (MSE), their future career interests, their preferences for particular career activities, and their likelihood to pursue a STEM career.

**Results:**

Analysis revealed that while older students had more knowledge about mathematics/science requirements for STEM careers, this knowledge was lacking overall. Also, students with higher MSE were more knowledgeable about STEM career requirements. Furthermore, students with higher MSE and STEM career knowledge were more likely to choose a STEM career. Students with greater interest in technical and scientific skills were also more likely to consider a STEM career than those who preferred career activities that involved practical, productive, and concrete activities.

**Conclusions:**

The results of this study show that students in middle school have a limited STEM career knowledge with respect to subject requirements and with respect to what sort of activities these careers involve. Furthermore, students with low MSE have a declining interest in STEM careers. Our data thus support the need to improve access to knowledge to facilitate students’ understanding of STEM careers and the nature of STEM work. Exposure of students to STEM careers can enhance their interest in pursuing careers involving science, technology, engineering, and mathematics.

## Background

Globally, youth vary considerably in their level of science, technology, engineering, and mathematics (STEM) career knowledge, their career interests, and their intentions of pursuing a STEM career. STEM career knowledge, defined as a student’s familiarity with a particular STEM career, varies considerably based on the school’s STEM career guidance. The level of STEM career knowledge an individual has will directly affect one’s intentions of pursuing a STEM career in the future (Compeau 2016; Nugent et al. 2015; Zhang and Barnett 2015). Without adequate knowledge, there is a risk that students will dismiss a STEM-based career path as a potential option for their future. Consequently, student interest in a particular STEM career will wane, which will negatively influence their desire to participate in activities that serve to increase STEM career knowledge and awareness. Indeed, interventions have shown that equipping students with STEM career knowledge early increases their motivation to take more science and mathematics courses in high school (Harackiewicz et al. 2012).

Students’ career interest and their preferred future career activities will also affect their intention of pursuing a STEM career. A key predictor of STEM career interest at the end of high school is interest at the start of high school (Sadler et al. 2012). However, the positive attitudes towards science identified in youth age 10 sharply declines by age 14 (Murphy and Beggs 2005; Tai et al. 2006); the junior high school years are typically ages 12–14 years. An extensive study in 2015, surveying 24,000 students, showed that occupational intentions change dramatically between the 9th and 11th grade and that the relationship between STEM intention and motivation is highly time-sensitive (Mangu et al. 2015).

Both STEM career knowledge and career interests are also influenced by society at large. These society influencers include role models that students are exposed to either in person or through the media, the individual students interact with on a daily basis such as teachers, family members, and peers, as well as students’ extracurricular experiences (Dabney et al. 2012; Harackiewicz et al. 2012; Nugent et al. 2015; Sahin et al. 2014; Sahin et al. 2015; Schumacher et al. 2009; Sjaastad 2012; Steinke et al. 2009; Zhang and Barnett 2015). Collectively, these influencing factors predict the self-efficacy (i.e., one’s belief in one’s ability) youth hold about their career options as well as their outcome expectancies (Mangu et al. 2015). Self-efficacy is considered a major predictor guiding the selection of majors during high school and post-secondary education (Heilbronner 2009; Kelly et al. 2013).

The grades 7 through 9 years (12–15-year-olds) are the key time period for influencing STEM career interest and for building this self-efficacy with respect to mathematics and science. Thus, it is during the junior high (middle) school age that a student’s beliefs about competency and interests begin to solidify (Simpkins et al. 2006). It is at this time that student engagement activities and career knowledge should be at its highest. Social cognitive career theory (Lent 2005) acknowledges and hypothesizes that career interests, choice, and personal goals form a complex human agency process that includes performance, self-efficacy, and outcome expectations. For example, self-efficacy is positively related to student academic performance and science self-efficacy has been shown to impact student selection of science-related activities, which impacts their ultimate success and helps maintain interests (Britner and Pajares 2006; Parker et al. 2014; Richardson et al. 2012).

Early interest in STEM topics is an excellent predictor for later learning and eventual career interests and choice (DeBacker and Nelson 1999). Contextual and individual variables influence these social cognitive variables including factors such as parental, teacher, and peer cultural expectations (Lent et al. 1994). Nugent et al. (2015) found support for the social cognitive career theory (Lent et al. 1994) as a framework for examining STEM learning and career orientation outcomes by providing a way in which to view the socio-contextual, motivational, and instructional factors that can impact youth STEM interests.

Although 88% of parents believe they can help guide their children’s learning, less than 28% actually discuss the value of a STEM education with their children (“Let’s Talk Science Canada Annual Report,” 2015). Recent studies have also indicated that junior high students have an unclear view about engineering (Compeau 2016; Karatas et al. 2011) and science (Masnick et al. 2010) yet these are critical years in which to build STEM interest. The present paper builds on our previous study (Franz-Odendaal et al. 2016) and explores students’ knowledge of STEM career mathematics/science requirements and their mathematics self-efficacy (MSE) and how these shape students’ career interests and preferred career activities. Differences among grade 7 and 9 students with respect to career interests and activities, and the likelihood of pursuing a STEM career will be examined. While gender differences are important because STEM stereotypes are heavily biased towards males, these differences are beyond the scope of the current study. This study will examine who, what, and how youth are influenced in STEM career choice.

This study captured five main areas of interest: student knowledge of mathematics and science requirements that lead to STEM careers, MSE, career interests, career activity preferences, and their correlation with the likelihood to consider pursuing a STEM career among youth. Based on the literature, the following research questions were developed to guide this research.

- RQ1: What is the correlation between grade level and students’ knowledge of high school requirements for STEM careers?
- RQ2: What is the correlation between MSE and students’ knowledge of high school requirements for STEM careers?
- RQ3: What is the correlation between MSE and students’ career interests and/or their preference for particular career activities?
- RQ4: What is the association between student preferences for career interests and preferred career activities with grade level?
- RQ5: What are the relationships between the following factors and the likelihood that students will choose a STEM career: grade level, MSE, student knowledge of mathematics/science requirements for post-secondary study for STEM careers, career interests and preferred career activities?

These research questions have not been explored in the context of Atlantic Canada, thus making this study relevant to the education system within Canada and globally.

## Methods

### The sample

Grade 7 and 9 students in the four Canadian Atlantic provinces (New Brunswick, Nova Scotia, Prince Edward Island, and Newfoundland) completed an online survey during their school hours. This research was approved by the university research ethics board. Permission to collect data in the schools was obtained from school board superintendents and parents. Schools were purposefully chosen from school families in geographic areas across Atlantic Canada. English and French language schools were included in the study. Data were weighted to ensure that the sample was representative by grade level, from each of the four Atlantic provinces. A total sample size of 1448 students was obtained across all four provinces in Atlantic Canada: New Brunswick (33%), Nova Scotia (38.4%), Prince Edward Island (6.5%), and Newfoundland-Labrador (22.1%). The sample was split almost evenly between grade 7 (48%) and grade 9 (52%). The sample was balanced with respect to gender (58% female to 42% male). Students ranged in age from 11 to 20 years with an average age of 13.5 years and a median age of 14 years (SD = 1.1). Grade 7 students had an average age of 12.6 years (SD = .6) with a median age of 13 years. Grade 9 students had an average age of 14.5 years (SD = .6) with a median age of 14 years.

### Measures

Five different measures were used in this study. These included measures of STEM career knowledge, MSE, career activity preferences, career interests, and likelihood to choose to pursue a STEM career. These measures were incorporated into the study based on earlier reviews that found that studies’ examining factors influencing career choice have been criticized for failing to account for the complexity of career choices and career decision-making (Patton and McMahon 2006) and for being too static in their view of career development (Hirshi 2011).

#### STEM career knowledge score

A STEM career knowledge (SCK) score was created to capture students’ knowledge about the requirements for high school mathematics and science in STEM careers. Students were presented with 12 STEM careers and asked to indicate whether they believed that the training for each of the careers required having taken mathematics or science in high school. Students could respond “yes” if they believed the career required high school mathematics or science based on their knowledge of the entrance requirements for Canadian colleges and universities. They could respond “no” if they believed that the career did not require high school mathematics or science, or they could choose “uncertain” if they were not sure that high school mathematics and/or science were required for that career. The list included careers students are commonly exposed to (such as veterinarian, pharmacist, and oral hygienist) as well as careers that are likely less familiar to them (such as mechanical engineer, geologist, and land surveyor). The list included mechanical engineer, computer hardware designer, pharmacist, medical technologist, geologist, veterinarian, oil industry engineer, physiotherapist, oral hygienist, nutritionist, land surveyor, and ophthalmologist. The list was provided to students in no particular order.

A score was calculated to capture students’ knowledge based on these responses. “Yes” responses were scored as “1,” “uncertain” responses as “0,” and “no” scored as “− 1.” The responses were then summed to obtain a basic SCK score per student. The SCK score was calculated only for students who had rated at least one third of the careers in the list. The SCK was validated using confirmatory factor analysis (CFA) and reliability analysis.

#### Mathematics Self-Efficacy Scale

In an attempt to offer a more complete perspective on the process of career decision-making, Hackett and Bertz (Hackett and Betz 1981) drew on the work of Bandura (1977) to introduce the concept of self-efficacy to the career development literature noting its potential to help understand the complexity of career decision-making such as the underrepresentation of women in traditional male-dominated career fields. Self-efficacy referred to the belief that a person had in their own ability to successfully perform a particular behavior based on their perception of their capability and the likelihood of their achieving success in that activity.

The second measure used in this analysis was a MSE scale. Students were asked to describe their experiences in mathematics by rating each of the following statements on a scale ranging from (1) Strongly disagree to (5) Strongly agree: I get good grades in mathematics; I learn quickly in mathematics; I look forward to my mathematics class; I feel tense doing mathematics problems; I feel helpless doing mathematics problems. Negatively phrased items were reverse-coded to maintain consistency in the MSE scale. Students’ responses to these five statements about their experiences in mathematics were then coded into dichotomies to create five separate measures. Dichotomies were created by assigning a value of “1” to those who were most comfortable with Likert scale ratings of 4 or 5 to the statements and assigning a value of 0 to those who were less confident and comfortable with Likert scale ratings of 1 through 3. These five measures were then summed to get a single MSE scale that would reflect higher measures for those who were the most confident and comfortable with mathematics. The MSE scale was validated using confirmatory factor analysis (CFA) and reliability analysis. The resulting MSE score ranged from 0 to 5. The MSE scale was then divided into two subgroups to create an MSE score for further analysis. The MSE score consisted of those with low MSE (scores of 0 through 3) and high MSE (scores of 4 or 5). The goal in using this breakout was to identify students who were the most comfortable and confident in their mathematics experiences.

#### Career activities and career interests

Social Cognitive Career Theory (SCCT) (Lent et al. 2010) has continued to evolve to include person and environmental and socio-demographic variables as well as interest and career choice models. The SCCT argues that people develop interests (actively likes and dislikes) largely on the basis of their beliefs about their self-efficacy and the outcomes their efforts could achieve. Ultimately, people become interested in activities they believe they can perform well. Therefore, people develop goals to pursue academic and career activities that are consistent with their interests as well as with their self-efficacy and outcome expectations (Sheu et al. 2010). Thus, career activities and career interests are highly correlated. For these reasons, student ratings of self-perceptions of their career interests, and also their career activities, were included.

The SCCT has been found to support self-efficacy and outcome expectations as significant predictors of interest, that interests partially mediate the relation of self-efficacy and outcome expectations, and that self-efficacy relates to outcome expectations across Holland’s (1997) broad occupational themes as utilized in the current study (Sheu et al. 2010). This alignment was felt to provide a rationale for the use of student ratings of interests, activities, and Holland’s broad occupational themes as a comprehensive way of gaining insight into the complexity of career decision-making of junior high students.

Hollands’ Theory of Career Choice and Development (Holland 1973) focused on six basic personality types: realistic (practical); investigative (analytical, curious); artistic (expressive, original); social (working/helping others); enterprising (goal oriented); and conventional (ordered). Individuals are not limited to one personality type and many exhibit characteristics on more than one type. Holland (1973) argued that everyone has career decisions to make at various stages of their lives. As well, he argued that everyone can serve as both a coach and/or a player in those decisions depending on their role, situation, and knowledge. Reflecting on the life stage, the environment, and the knowledge one has of their own particular type of preferred approach to life plus knowledge of the interaction among a variety of factors such as the cultural, social, academic, and family influences on the decisions that each individual makes about their life career. These are not perfect, single, nor static events and depend on self and other perceptions of a wide range of factors. However, at a point in time, they represent what each person conceptualizes as a satisfying career for them. Holland (1973) argued that his theory of careers was really intended to help practitioners, researchers, and students in education and social science to address a fuller understanding of vocational choice and to be helpful in professional counseling. Miller (1998) stated that Holland’s theory can be used to help individuals explore career choices. More recently, Olitsky (2014) used Holland’s theory of career and educational choice when researching the earnings of STEM majors, indicating that the underlying theory is still relevant. Since career interests and career activities are highly correlated, they were measured separately.

The third measure used in this analysis was a ranking of the preferred career activities using Holland’s Theory of Career Choice and Development (1973). Students were asked to rank six different career activities from (1) Most favorite to (6) Least favorite. Each of the career activities was then analyzed based on the percentage of students who rated it in their top 2 favorites. The career activities studied in this research included the following: (1) artistic, unusual, and creative activities; (2) working on practical, productive, and concrete activities; (3) taking responsibility, providing leadership, and convincing others; (4) things being organized into routines and having an order; (5) learning by reading, study, analysis, or investigation; (6) helping others and being concerned for the welfare of others.

The fourth measure used in this analysis was a ranking of career interests also based on Holland’s Theory of Career Choice and Development (Holland 1973). Students were asked to rank six different career interests from (1) Most favorite to (6) Least favorite. Each of the career interests was then analyzed based on the percentage of students who rated it in their top 2 favorites. These interests were (1) working with people; (2) creative skills and expression; (3) technical and scientific skills; (4) manual and mechanical skills; (5) leading, persuading, and directing others; and (6) routines and adhering to standards of performance.

#### Likelihood to pursue a STEM career

The final measure used in this analysis was the likelihood that students would consider choosing a STEM career in their future. Students were asked how likely they would be to choose a career that is science-related (including science, engineering, health, or technology). Likelihood was measured using the following Likert scale: (1) Very unlikely, (2) Somewhat unlikely, (3) Somewhat likely, and (4) Very likely. This scale was recoded into a dichotomous variable for use in bivariate logistic regression: Students who were somewhat likely or very likely to choose a STEM career were coded as “1,” and those who were somewhat unlikely or very unlikely to choose a STEM career were coded as “0.”

### Data analysis

Data were analyzed using the SPSS software (IBM Corp 2013). Descriptive statistics were used to provide an overall analysis of the data. Various statistical tests were selected based on the level of data measurement and data distributions (McDaniel et al. 2014; Hair Jr. et al. 2010). *t* tests were used to explore differences in average ratings between groups. Chi-square was used to analyze associations between nominal and ordinal variables. Analysis of variance (ANOVA) was used to evaluate significant differences between average ratings and measures involving categorical variables with more than two response levels (McDaniel et al. 2014). Logistic regression was used to explore research questions involving interval and ratio-scaled variables (Hair Jr. et al. 2010). Brown-Forsyth exact tests were used with ANOVA to compensate for violations of homogeneity of variance (IBM Corp 2013). Bonferroni post hoc tests were used to detect significant differences between groups for significant ANOVA results (IBM Corp 2013; Hair Jr. et al. 2010). Data were weighted to reflect the population of students by grade level and province across Atlantic Canada.

Bivariate logistic regression was conducted to explore the relative contribution of the following factors on the likelihood that students would choose a STEM career: SCK score, MSE score, grade level, career interests, and career activities. Grade level, career interest, and career activities were coded as dichotomies for the regression analysis as follows: grade level (grade 9 = 1, grade 7 = 0), career interests (rated in top 2 favorites = 1, not rated in top 2 favorites = 0), career activities (rated in top 2 favorites = 1, not rated in top 2 favorites = 0).

Three regressions were created to explore the research questions. The first analysis regressed grade level, SCK score, and MSE score against the likelihood to pursue a STEM career. Two more regressions were conducted: one to regress career activities and a second to regress career interests against the likelihood to pursue a STEM career as a dependent variable. Measures for career activity and career interests showed a high level of multicollinearity between the two sets of variables. Separating these predictors into two different regressions eliminated problems with multicollinearity.

## Results

We first describe the results for each of the measures used in this study and then answer our research questions (RQ1–5).

### Student knowledge of mathematics and science requirements for STEM careers

We assessed student’s knowledge of high school requirements for STEM careers, by asking students to indicate whether a career required mathematics and/or science (Table 1). Mechanical engineer was noted by 71.4% of students as having a high school mathematics/science requirement. Two careers (land surveyor and ophthalmologist) were noted by less than half of the students as requiring high school mathematics or science. Five careers were classified as requiring mathematics and science by 65.6 to 68.2% (veterinarian, geologist, medical technologist, pharmacist, computer hardware engineer). Four of the careers were listed as requiring mathematics and science by 51.8 to 58.6% of the students (nutritionist, oral hygienist, physiotherapist, oil industry engineer). What is notable in the students’ responses are that most students seemed confident of their career classification in that they answered “yes” or “no” and not the option of “uncertain,” indicating that they were confident in their choice. The percentage of students saying that they were uncertain if a career required mathematics or science for post-secondary study was low and ranged from 12.5 to 32.6% across all of the careers with half of the uncertain responses ranging from 12.5 to 13% of students. Table 1 shows the results of high school mathematics/science requirements for STEM careers.

**Table 1:** Classification of careers as requiring mathematics and/or science by students in Atlantic Canada

Career	Does the career require high school mathematics and/or science for entry into university?			
	Yes (%)	No (%)	Uncertain (%)	Number
Mechanical engineer	71.4	16.1	12.5	1250
Computer hardware designer	68.2	17.3	14.5	1259
Pharmacist	67.9	18.6	13.5	1257
Medical technologist	67.1	18.8	14.0	1243
Geologist	67.0	20.0	13.0	1249
Veterinarian	65.6	19.3	15.1	1243
Oil industry engineer	58.6	21.3	20.1	1262
Physiotherapist	54.8	24.1	21.1	1254
Oral hygienist	53.1	25.7	22.5	1252
Nutritionist	51.8	25.7	22.5	1247
Land surveyor	48.2	27.7	24.1	1232
Ophthalmologist	46.6	20.9	32.6	1223

Sample size = 1448. Data weighted by grade and province

Students’ responses were summed to obtain an overall SCK score. A factor analysis of the career ratings was used to ensure it was unidimensional, and reliability of the score was measured using Cronbach’s alpha. The confirmatory factor analysis was statistically significant (KMO = .961, *p* < .01). Cronbach’s alpha was .95 which meets the criterion for reliability.

**Fig. 1:**
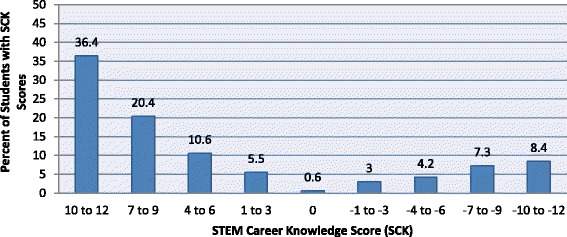
STEM career knowledge (SCK) score

The SCK score ranged from − 12 to + 12, with an average score of 4.6 (SD = 7.6; Fig. 1). The average SCK score was low, indicating a lack of familiarity with the mathematics and/or science requirements of STEM careers. Approximately 8% of students did not correctly classify any of the careers as having a high school mathematics and/or science requirement. Only 36.4% of students had high SCK scores having correctly classified 10 to 12 careers. The top quartile of students scored 11 or better while the bottom quartile scored 0 or less than 0 out of the 12-point score. A summary of the SCK score is in Fig. 1.

Overall, these results suggest that STEM career knowledge is limited among middle school students. Results also reveal that students seem to be unaware of their limited knowledge regarding STEM career preparation.

### Students’ mathematics self-efficacy

**Fig. 2:**
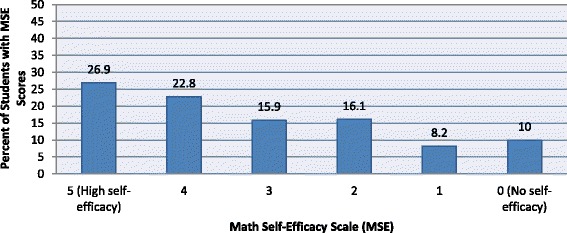
Mathematics Self-Efficacy (MSE) Scale

In order to determine whether MSE was correlated with students STEM career knowledge (RQ2) and/or between MSE and career interests and/or preferred career activities (RQ3), we first determined the MSE scale for the cohort. The MSE scale ranged from 0 (No self-efficacy) to 5 (High self-efficacy). The distribution of the Math Self-Efficacy Scale is shown in Fig. 2.

A confirmatory factor analysis of the measures in the MSE scale indicated that it was unidimensional and reliable. The factor analysis was statistically significant (KMO = .698, *p* < .01). Cronbach’s alpha was .72 which is acceptable for a scale analysis. These results suggest that over half of the students had a relatively high MSE and about one third of students had low MSE.

### Career activities and interests

In order to assess students’ preferred career activities and their career interests, students were asked to select their favorites. Students were presented with a list of six career activities and asked to indicate which activities were in their top 2 favorites. These measures were recoded into dichotomies for further analysis. There was a very even spread of students rating career activities in their top 2 favorites, ranging from 32.1 to 45.9%. Most of the activities were listed in their top 2 favorites by about one third of the students. The results revealed that artistic, unusual, and creative activities were most commonly listed in the top 2 favorite career activities. The career activity with the lowest rating was helping others and being concerned for their welfare. The results are summarized in Table 2.

**Table 2:** Career activities rated by grade 7 and 9 students in Atlantic Canada as being in the top 2 favorites

Career activity	Percent	Number
Artistic, unusual, creative activities	45.9	548
Practical, productive, concrete activities	38.5	457
Taking responsibility, providing leadership, convincing	36.3	430
Organized into routines and having order	34.7	410
Reading, study, analysis, investigation	33.7	390
Helping others and being concerned for welfare	32.1	374

Sample size = sizes range from 1182 to 1199 across career activities. Data weighted by grade and province

Students were also presented with a list of six career interests and asked to indicate which career interests were in their top 2 favorites. These measures were recoded into dichotomies for further analysis. The percentage of students rating career interests as their top 2 favorites ranged from 21.9% for routines and adhering to standards to working with people at 49.8%. The results are summarized in Table 3.

**Table 3:** Career interests rated by grade 7 and 9 students in Atlantic Canada as being in the top 2 favorites

Career interest	Percent	Number
Working with people	49.8	593
Creative skills and expression	41.2	487
Technical and scientific skills	39.4	463
Manual and mechanical skills	32.9	387
Leading, persuading, and directing others	32.7	384
Routines and adhering to standards	21.9	255

Sample size = 1448. Data weighted by grade and province

### Likelihood of choosing a STEM career

Next, we assessed whether students were interested in pursuing a STEM career. Nearly 70% percent of students surveyed revealed that they were either somewhat likely or very likely to pursue a STEM career. On a scale of (1) Very unlikely to (4) Very likely, an average rating of 2.9/4 indicated that students were somewhat likely to pursue a STEM career. The results appear in Table 4.

**Table 4:** Likelihood of choosing a STEM career

	Percent	Number
(4) Very likely	32.7	444
(3) Somewhat likely	36.8	499
(2) Somewhat unlikely	15.9	215
(1) Very unlikely	14.6	197
Total	100.0	1356

Sample size = 1356. Mean = 2.9, Median = 3.0, SD = 1.0. Data weighted by grade and province

### The association of grade level and STEM career knowledge

The first research question (RQ1) explored the correlation of grade level and STEM career knowledge. There was a statistically significant difference in the average SCK score by grade, with grade 9 students scoring higher than grade 7 students (5.7 vs 3.3, *t* = − 5.69, df = 1209.7, *p* < .01). While it is good to see that students appear to acquire more knowledge of STEM career requirements in middle school grades, it is concerning that students in grade 9 still had a low average SCK score since this is the year in which students begin to choose subject classes in Atlantic Canada. This indicates that more work is needed to ensure students have the correct information about STEM career requirements in time for them to make informed decisions about high school course selection.

### Correlation between mathematics self-efficacy and the STEM career knowledge

The second research question (RQ2) focused on whether there is a correlation between students with higher MSE and knowledge of STEM career requirements. An analysis of variance revealed that students with high self-efficacy (MSE scale = 4 and 5) had significantly higher SCK scores than students who did not score as highly in the MSE scale (BF = 8.7, df = 5, *p* < .01). Students with high MSE had a SCK score of 6.6 out of 12, while students with lower MSE scores had average SCK scores ranging from 2.8 to 4.8. The results are shown in Table 5.

**Table 5:** The correlation between mathematics self-efficacy (MSE) on STEM career knowledge (SCK) scores

Mathematics Self-Efficacy Scale	STEM career knowledge score		
	Average	Std. deviation	Number
5	6.6	6.6	365
4	4.8	7.3	304
3	3.6	7.9	194
2	3.0	8.3	197
1	2.8	8.5	101
0	3.3	7.5	105

Sample size = 1266. Data weighted by grade and province. The SCK score for students with high MSE is significantly higher than the average ratings for any of the lower self-efficacy scores (Brown-Forsyth exact test = 8.7, df = 5/824, *p* < .01). Minimum = − 12, maximum = + 12

These results for RQ2 show that students who report more confidence and comfort in mathematics tend to be more knowledgeable about mathematics/science requirements for STEM careers. This is a correlation only and cannot be interpreted as a causal relationship since survey data cannot be used to measure causality.

### Correlation between mathematics self-efficacy and students’ preferred career activities and their career interests

Our third research question (RQ3) explored whether there was a correlation between MSE and students’ career interests and preferred career activity. There were statistically significant differences by students’ preferred career activities for the MSE scale. MSE scale totals were sorted into two groups to create an MSE score for further analysis. Those with low MSE scale totals (0 through 3) were assigned an MSE score of 0, and those with high MSE scale totals (4 and 5) were assigned an MSE score of 1. A chi-square analysis revealed that only one career activity differed significantly based on students’ MSE scores. Reading, study, analysis, and investigation was listed in the top 2 favorites for career activities by 36.5% of students who had high MSE scores (between 4 and 5) when compared to 28.4% of students with low MSE (0 through 3) (*χ*^2^ = 7.979, df = 1, *p* < .01). The remaining career activities did not differ significantly based on students’ MSE. The results are summarized in Table 6.

**Table 6:** Analysis of students’ favored career activities by mathematics self-efficacy (MSE)

	Mathematics self-efficacy score	
Career activity listed in top 2 favorites	0 to 3 Low self-efficacy	4 to 5 High self-efficacy
Helping others and being concerned for welfare	146 (31.9%)	200 (57.8%)
Artistic, unusual, creative activities	230 (49.6%)	286 (45.0%)
Practical, productive, concrete activities	178 (38.7%)	247 (38.7%)
Taking responsibility, providing leadership, convincing	166 (35.9%)	235 (37.1%)
Organized into routines and having order	145 (31.2%)	233 (36.8%)
Reading, study, analysis, investigation^	131 (28.4%)	232 (36.5%)

Differences are statistically significant at the .01 level or better (^). Data weighted by grade and province. Sample size varies from 1088 to 1100 for each activity. Results show the percent within each MSE group (low or high MSE) that rated the career activity shown in their top 2 favorites. Percentages do not sum to 100%

These results show that most of the preferred career activities had no correlation at all with MSE scores. However, reading, study, analysis, and investigation are the hallmarks of a mathematics-, science-, or technology-based activity. Therefore, it is reasonable that students who are confident and comfortable with mathematics would also enjoy reading, study, analysis, and investigation.

In order to explore whether there is a correlation between MSE and student’s career interests, a chi-square analysis was conducted. The chi-square analysis revealed that only one career interest differed significantly based on students’ MSE score. This career interest was technical and scientific skills. This career interest was listed in the top 2 favorites for career activities by 43.8% of students with high MSE score (between 4 and 5) compared to 36.0% of students with low MSE score (0 through 3) (*χ*^2^ = 6.558, df = 1, *p* = .01). The remaining career interests did not differ significantly based on students’ MSE scores. As with the results for career activities, these results show that most of the career interests were not significantly correlated with MSE and all of the career interests were rated in the top 2 favorites by less than half of the students. It is reasonable that students who are confident and comfortable with mathematics would also be interested in careers involving technical and scientific skills. The results are summarized in Table 7.

**Table 7:** Analysis of students’ favored career interests by mathematics self-efficacy (MSE)

	Mathematics self-efficacy score	
Career interest listed in top 2 favorites	0 to 3 Low self-efficacy	4 to 5 High self-efficacy
Working with people	215 (46.8%)	328 (51.1%)
Technical and scientific skills^	159 (36.0%)	280 (43.8%)
Creative skills and expression	195 (43.5%)	267 (41.5%)
Manual and mechanical skills	150 (33.6%)	205 (32.2%)
Leading, persuading, and directing others	139 (31.3%)	205 (32.1%)
Routines and adhering to standards	104 (23.8%)	130 (20.4%)

Differences statistically significant at the .01 level (^). Data weighted by grade and province. Sample sizes varied from 1073 to 1101 by career interest. Results show the percent within each MSE group (low or high MSE) that rated the career interest shown in their top 2 favorites. Percentages do not sum to 100%

### The correlation between grade level and students’ career interests and preferred career activities

The fourth research question (RQ4) addressed whether grade level was associated with student preferences for career interests and activities. There were statistically significant differences by grade regarding some of the career interests, thereby satisfying the first part of the fourth research question. More grade 7 than grade 9 students listed manual and mechanical skills in their top 2 favorites (36.5 vs 29.4%, *χ*^2^ = 6.84, df = 1, *p* < .01), as well as creative skills and expression (45.4 vs 37.0%, *χ*^2^ = 8.73, df = 1, *p* < .01). More grade 9 than grade 7 students ranked “working with people” in their top 2 favorites (52.8 vs 46.8%, *χ*^2^ = 4.21, df = 1, *p* < .05). These results are summarized in Table 8.

**Table 8:** Analysis of students’ favored career interests by grade

Career interest listed in top 2 favorites	Grade 7		Grade 9	
	%	Number	%	Number
Working with people^	46.8	277	52.8	316
Technical and scientific skills	40.3	236	38.6	227
Creative skills and expression^	45.4	268	37.0	220
Leading, persuading and directing others	31.7	185	33.7	199
Manual and mechanical skills^	36.5	214	29.4	173
Routines and adhering to standards	19.8	115	24.1	140

Sample size ranges from 255 to 593 across career interests. Differences are statistically significant at the .05 level or better (^). Data weighted by grade and province

More grade 7 than grade 9 students listed practical, productive, and concrete activities in their top 2 favorites (42.1 vs 34.7%; *χ*^2^ = 6.9, df = 1, *p* < .01). More grade 9 than grade 7 students rated helping others and being concerned for their welfare in their top 2 favorite career activities (34.9 vs 28.6%; *χ*^2^ = 5.4, df = 1, *p* < .05) as well as having things organized into routines and having order (39.4 vs 29.4%; *χ*^2^ = 13.2, df = 1, p < .01). There were no statistically significant differences by grade level for the other career activities studied.

This trend is similar to that emerging in the analysis of career interests. In general, students in the higher grade focused more on activities involving helping others and being less attracted to careers that involved practical applications or routines. The results are summarized in Table 9.

**Table 9:** Analysis of students’ favored career activities by grade

Career activity listed in top 2 favorites	Grade 7		Grade 9	
	%	Number	%	Number
Artistic, unusual, creative activities	47.2	283	44.5	265
Organized into routines and having order^	29.4	173	39.4	236
Taking responsibility, providing leadership, convincing	36.4	217	36.0	213
Helping others and being concerned for welfare^	28.6	170	34.9	204
Practical, productive, concrete activities^	42.1	249	34.7	207
Reading, study, analysis, investigation	33.6	199	32.0	191

Sample size ranges from 374 to 548 across career activities. Differences statistically significant at the .05 level or better (^). Data weighted by grade and province

### The correlations between students’ STEM career knowledge, mathematics self-efficacy, and grade level on their likelihood to choose a STEM career

The fifth research question (RQ5) focused on how several aspects might relate to students’ likelihood of choosing a STEM career. These areas included grade level, MSE, knowledge of STEM careers, and preferences for various career interests and activities.

First, a logistic regression was conducted to determine whether or not grade level, STEM knowledge, and MSE score were associated with students’ likelihood to pursue a STEM career. The hypothesized regression model was likelihood of choosing a STEM career (ODDS) = *f*(grade level, STEM knowledge score, MSE score). A test of the full regression model against an intercept-only model was statistically significant (*χ*^2^ = 76.85, df = 3, *p* < .01). The regression was strong with a McFadden’s *R*^2^ = .85.

The regression analysis correctly classified 70.6% of all cases and 95.3% of those who were likely to choose a STEM career. The regression revealed that students with stronger SCK scores were marginally more likely to pursue a STEM career than were students with weaker SCK scores (odds ratio = 1.04, probability = .51). However, students with high MSE scores were 1.3 times more likely to pursue a STEM career than were those who had lower MSE scores (probability = .56). Grade level was not a statistically significant predictor of the likelihood of pursuing a STEM career.

These results showed that students’ knowledge of STEM careers and their self-efficacy in mathematics were statistically significant factors in the likelihood that they would pursue a STEM career, while STEM career knowledge was a modest contributor. Also, students in grade 9 were not more likely to pursue a STEM career than were students in Grade 7. However, research has shown that occupational intentions change dramatically between 9th and 11th grades and the relationship between STEM intention and motivation is very time-sensitive (Mangu et al. 2015, p.55). The results are summarized in Table 10.

**Table 10:** Bivariate logistic regression of the correlations between grade level, STEM knowledge score and mathematics self-efficacy by students’ likelihood to pursue a STEM career

	B	Wald *χ*^2^	Sig.	Exp(B) (ODDS)	Probability
STEM career knowledge score	.042	25.053	.000	1.043	.51
Mathematics self-efficacy score	.236	34.146	.000	1.266	.56
Grade 9	.232	3.073	.080	1.261	.56
Constant	− .145	.964	.964	.857	–

Sample size = 1215. Data weighted by grade and province

These regression results reveal that individual student characteristics, MSE, and SCK are better predictors of the likelihood to pursue STEM careers than student grade level. Individual strengths and weaknesses, as well as students’ knowledge and competency, are better indicators of future career paths than grade level.

### The correlation between students’ career interests and their likelihood to pursue a STEM career

A second logistic regression was conducted to explore whether or not students’ preferred career interests was correlated with their likelihood to pursue a STEM career. Six career interests were explored in the analysis. The hypothesized regression model was likelihood of choosing a STEM career (ODDS) = *f*(manual and mechanical skills; technical and scientific skills; creative skills and expression; working with people; leading, persuading, and directing others; routines and adhering to standards). A test of the full regression model against an intercept-only model was statistically significant (*χ*^2^ = 119.94, df = 6, *p* < .01). The regression was reasonably strong with a McFadden’s *R*^2^ = .73. The regression analysis correctly classified 72% of all cases and 96.6% of those who were likely to choose a STEM career.

The regression revealed that students who rated technical and scientific skills in their top 2 favorite career interests were 5.4 times more likely to pursue a STEM career (probability = .84). Students who rated working with people in their top 2 favorites were 1.5 times more likely to pursue a STEM career (probability = .61). Students who rated creative and expressive skills in their top 2 favorite career interests were less likely to pursue a STEM career than those who rated creative and expressive skills highly. Their odds of pursuing a STEM career were only .70 of those who did not rate creativity and expressiveness among their favorite career interests. Their probability of pursuing a STEM career was .41. The remaining career interests were not statistically significant predictors of the likelihood of pursuing a STEM career (manual or mechanical skills; leading, persuading, or directing others; routines and adhering to standards). These results provide evidence for the fifth research question in that three out of the six career interests measured did have a statistically significant correlation with the likelihood that a student would consider pursuing a STEM career. The results are summarized in Table 11.

**Table 11:** Bivariate logistic regression of the correlation between career interests and students being very likely to pursue a STEM career

Career interests listed in top 2 favorites	B	Wald *χ*^2^ (df = 1)	Sig.	Exp(B) (ODDS)	Probability
Technical and scientific skills	1.687	83.115	.000	5.402	.84
Working with people	.431	7.657	.006	1.538	.61
Creative skills and expression	− .356	5.309	.021	.700	.41
Routines and adhering to standards	.247	1.905	.167	1.280	.56
Manual and mechanical skills	− .16	.825	.364	.853	.46
Leading, persuading, and directing others	.073	.200	.655	1.076	.52
Constant	.335	2.621	.105	1.397	–

Sample size = 1126. Data weighted by grade and province

These results indicate that student preference for technical and scientific skills and careers involving working with people enhance the likelihood of pursuing a STEM career, while students who prefer careers involving creative skills and expression are less likely to do so. While a focus group could better explore the students’ preferences for creativity and creative careers, this level of detail is not possible in large sample survey-based research and is outside of the scope of this study. Other career interests that focus on mechanical, manual, or routine activities, or those involving leadership, do not predict the likelihood of students pursuing a STEM career and are not significantly correlated with STEM career choice.

A third logistic regression analysis was conducted to determine whether students’ career activity preferences were correlated whether or not they were likely to pursue a STEM career. Six career activities were explored in the analysis. The hypothesized regression model was likelihood of choosing a STEM career (ODDS) = *f*(practical, productive, concrete activities; reading, study, analysis, and investigation; artistic, unusual, and creative activities; taking responsibility, providing leadership, and convincing; and helping others and being concerned for their welfare). A test of the full regression model against an intercept-only model was statistically significant (*χ*^2^ = 32.883, df = 6, *p* < .01). The regression was reasonably strong with a McFadden’s *R*^2^ = .78. The regression analysis correctly classified 72% of all cases, and 100% of those who were likely to choose a STEM career.

The regression revealed that students who preferred career activities involving reading, study, analysis, and investigation were 1.8 times more likely to pursue a STEM career (probability = .65) than those who did not prefer such activities. Students’ rating career activities involving routines and having an order were 1.5 times more likely (probability = .60) to pursue a STEM career than those who did not prefer such activities, while students with preferences for practical, productive, and concrete career activities were 1.5 times more likely to pursue a STEM career (probability = .60) compared to those who did not prefer such activities. The remaining career activities were not statistically significant predictors of the likelihood to pursue a STEM career (artistic, unusual, and creative activities; taking responsibility, providing leadership, and convincing others; helping others and being concerned for their welfare). These results revealed three out of the six career activities measured did have a statistically significant correlation with the likelihood that a student would consider pursuing a STEM career. The results are summarized in Table 12.

**Table 12:** Bivariate logistic regression of the influence of career activity preferences on students being very likely to pursue a STEM career

Career activity listed in top 2 favorites	B	Wald *χ*^2^ (df = 1)	Sig.	Exp(B) (ODDS)	Probability
Reading, study, analysis, investigation	0.606	14.49	0.000	1.834	0.65
Organized into routines and having order	0.412	7.301	0.007	1.51	0.60
Practical, productive, concrete activities	0.389	6.518	0.011	1.475	0.60
Helping others and being concerned for welfare	0.179	1.441	0.23	1.197	0.54
Taking responsibility, providing leadership, convincing	0.143	0.915	0.339	1.154	0.54
Artistic, unusual, creative activities	− 0.091	0.399	0.528	0.913	0.48
Constant	0.42	5.466	0.019	1.522	–

Sample size = 1129. Data weighted by grade and province

These results stand in contrast to those for students’ career interests and the likelihood of pursuing a STEM career. Unlike the career interest analysis, students seeking routine career activities are more likely to pursue a STEM career. Also, students who ranked career interests involving helping others were more likely to pursue STEM careers, but this analysis showed that student preference for career activities involving helping others and being concerned for their welfare was not a statistically significant indicator of their likelihood to pursue a STEM career. Further, these results differ somewhat in terms of students’ preferences for practical activities. While career interests involving manual or mechanical skills were not statistical indicators of the likelihood of pursuing a STEM career, career activities involving practical, productive, and concrete activities were statistically significant. The career activity involving reading, study, analysis, and investigation was also statistically linked to students’ likelihood to pursue a STEM career, which seems reasonable given that such activities are at the heart of many STEM careers.

## Discussion

Youth vary widely in their career knowledge, interest, and intentions. Factors investigated in the present study examined STEM career knowledge, MSE, career activities, career interests, and the likelihood of students to pursue a STEM career.

### Knowledge and self-efficacy

Results of the present study align with recent findings by Compeau et al. (2016), Nugent et al. (2015), and Zhang and Barnett (2015) show that self-efficacy along with knowledge of STEM careers are significant factors in whether or not adolescents pursue STEM careers. Findings also indicated that career knowledge is limited among middle school students and students seem to be unaware of their limited knowledge regarding STEM preparation. While approximately 70% of students reported that mathematics was an important requirement for a career in mechanical engineering, computer hardware design, and pharmacy, 50% or less were aware that it was also important in careers for ophthalmology, land surveyor, nutrition, and oral hygienist.

The issue of self-efficacy takes on particular significance as students progress through high school. Previous research by Murphy and Beggs (2005), Heilbronner (2009), and Mangu et al. (2015) have noted how young women have a lower self-efficacy in STEM during high school years. Previous research has also shown that interest in STEM and motivation to pursue STEM activities tends to wane over time for all high school students. The results of the current study agree with earlier findings that lower levels of MSE exist; we found approximately 34% of participants had low MSE scale totals. These findings raise concerns about the combined effects of students’ low MSE and their declining interest in STEM from early through to later grades and on the numbers of graduating high school students who will be inclined to choose a STEM career.

### Career activities and interests

Results of the current study demonstrated that students in grades 7 and 9 had a broad range of favorite career activities with the majority (approximately 46%) stating that their strongest preference was for artistic and creative types of activities. Also, all of the possible activities were selected by at least one third of the group. Interestingly, approximately 50% of participants selected their career interest as being “working with people,” but relative to career activities, only one third of participants selected “helping people.” However, this is not surprising given that one can have an interest but may not want to have a career working in that activity. For example, one may be interested in art, but have no interest, or lack sufficient talent, to pursue a career in the field (Holland 1973). Also, middle school students may not be able to discriminate between the nuances between career activities and interests in the way that older students and young adults would. Although a focus group study may be able to further elucidate this issue, this is beyond the scope of the current study.

Working with others and participating in creative types of activities are important findings that relate to current issues in education in Canada. A recent study (Ayar and Yalvac 2016) found that many STEM careers are team-based, creative, and require technical, scientific, and problem solving skills. However, in Canada, many post-secondary programs continue to focus more on memorizing and replicating science content knowledge. Further study of this possible implication would be worthwhile.

### Likelihood of choosing a STEM career

While approximately 70% of participants stated they were likely to choose a STEM career, 30% were less likely to do so. Not all students have the financial means to pursue a career interest. In addition, career interest and motivation are highly time sensitive (Mangu et al. 2015, p.55). Considering findings from studies such as Ayar and Yalvac (2016), as well as what we know about the decline in interest in STEM careers as students mature, these results suggest that there is room for increasing awareness, STEM career interest, and providing for better knowledge acquisition in the area of STEM careers. As well, our data suggests that alternative ways of teaching and evaluating STEM courses should be considered. Perhaps a greater emphasis on authentic means of teaching and evaluating STEM content that involves collaboration, problem solving, and application of STEM knowledge might serve to engage learners in more meaningful ways, thereby enabling continued motivation and interest in STEM careers as students progress through secondary and post-secondary education. Does a higher MSE lead students to consider pursuing STEM careers and lead them to becoming more informed about the career requirements or do students who have higher knowledge of STEM careers become more competent in mathematics? Are these factors simply correlational and reflect students who are high in both measures or low in both measures? While these questions cannot be answered in this research, it is interesting to note that MSE may play a role, or be a leading indicator, for STEM career knowledge.

### Influence of mathematics self-efficacy on career knowledge, interest and activities

Our results indicate that while there is a relationship between career knowledge and MSE, we did not find a relationship among career interests or activities with MSE. The assumption that having a positive sense of mathematics skill would correlate with STEM career interests and activities was not supported. Follow-up research involving interviews with participants about their understanding of career interests, activities, and MSE, would provide more an in-depth understanding. Based on findings by Simpkins et al. (2006) it was expected that there would be a relationship among interests, activities and MSE as their findings indicated that in junior high beliefs about competency and interests begin to solidify. Further research may help to uncover reasons for not seeing such a correlation in this analysis.

### Influence of grade level on STEM career knowledge, interest, and activities

Results indicate that there were significant differences between grade 7 and grade 9 students in the present study relative to STEM career knowledge. Overall, students in grade 9 were more knowledgeable than grade 7 students about STEM careers. The differences formed an interesting and consistent pattern that more grade 7 students expressed interest in manual and mechanical skills than grade 9 students who tended to have more interest in working with people. Further, more grade 7 students expressed interests in practical and concrete types of activities while more grade 9 students expressed interests in helping people and being concerned for their welfare. Reasons for this shift are not clearly understood. As noted by Lent (2005), career interest, choice, and personal goals form a complex chain involving performance, self-efficacy, and outcome expectations. As well, socio-cultural factors also need to be considered along with opportunity for exposure (Fouad and Smith 1996; Kuncel et al. 2005; Lent et al. 1994). As with the relationship among self-efficacy, knowledge, interests, and activities, in-depth research involving student interviews may result in greater understanding of the reasons for these shifts and their impact on later careers.

### Factors influencing positive statements involving the likelihood of choosing a STEM career

Regression analyses revealed that participants with stronger STEM career knowledge were slightly more likely to pursue a STEM career and that students with higher MSE scores were also slightly more likely to choose a STEM career. Also, grade level was not a differentiating factor, which was anticipated given the small distance between the experiences of grade 7 versus grade 9 students. As noted in many previous studies (Lent et al. 1994; Kuncel et al. 2005), knowledge of STEM careers and self-efficacy in mathematics are statistically significant factors in the likelihood that participants will pursue STEM careers.

Interest in technical and scientific skills is a strong predictor of the likelihood of pursuing a STEM career with those who indicated a preference for technical and scientific skills being 5.4 times more likely to indicate the likelihood of choosing a STEM career compared to those who rated working with people as their stronger interest. Indeed, preferences for practical, productive, and concrete activities also indicated a stronger likelihood of pursuing STEM careers than those who do not prefer such activities. Implications of these findings point to improving methods for providing information on the skills and nature of the work in STEM careers particularly in fields such as engineering and technology (which have an important focus on team work, problem solving, and creativity) as well as on technical and scientific skills.

## Conclusions

Overall, results of the present study show that career knowledge is limited among middle school students and that they have a declining interest in STEM and have low MSE scores. Students are interested in careers that involve a wide variety of activities but do not appear to relate these activities to STEM careers. Our results point to the importance of finding and expanding on ways to increase authentic learning opportunities in secondary school in Atlantic Canada such that students are better able to participate in collaboration, problem solving, and the application of scientific knowledge in their classes. Such learning opportunities would ensure that students have access to more information on the actual nature of work in the STEM field and what is required to pursue these careers. This strategy would also serve as a motivator to those who are not aware that STEM careers involve people skills, creativity, and problem solving.

## Abbreviations

CFA: Confirmatory factor analysis
MSE: Mathematics self-efficacy
SCK: STEM career knowledge score
STEM: Science, technology, engineering, and mathematics
